# Phosphoprotein Phosphatase 1 Is Required for Extracellular Calcium-Induced Keratinocyte Differentiation

**DOI:** 10.1155/2016/3062765

**Published:** 2016-06-02

**Authors:** Chandrama Shrestha, Yuanyuan Tang, Hong Fan, Lusha Li, Qin Zeng, Sally D. Pennypacker, Daniel D. Bikle, Zhongjian Xie

**Affiliations:** ^1^Institute of Endocrinology and Metabolism, The Second Xiangya Hospital of Central South University, Changsha, Hunan 410011, China; ^2^Endocrine Unit, Veterans Affairs Medical Center, University of California, San Francisco, CA 94121, USA

## Abstract

Extracellular calcium is a major regulator of keratinocyte differentiation in vitro and appears to play that role in vivo, but the mechanism is unclear. We have previously demonstrated that, following calcium stimulation, PIP5K1*α* is recruited by the E-cadherin-*β*-catenin complex to the plasma membrane where it provides the substrate PIP2 for both PI3K and PLC-*γ*1. This signaling pathway is critical for calcium-induced generation of second messengers including IP3 and intracellular calcium and keratinocyte differentiation. In this study, we explored the upstream regulatory mechanism by which calcium activates PIP5K1*α* and the role of this activation in calcium-induced keratinocyte differentiation. We found that treatment of human keratinocytes in culture with calcium resulted in an increase in serine dephosphorylation and PIP5K1*α* activation. PP1 knockdown blocked extracellular calcium-induced increase in serine dephosphorylation and activity of PIP5K1*α* and induction of keratinocyte differentiation markers. Knockdown of PLC-*γ*1, the downstream effector of PIP5K1*α*, blocked upstream dephosphorylation and PIP5K1*α* activation induced by calcium. Coimmunoprecipitation revealed calcium induced recruitment of PP1 to the E-cadherin-catenin-PIP5K1*α* complex in the plasma membrane. These results indicate that PP1 is recruited to the extracellular calcium-dependent E-cadherin-catenin-PIP5K1*α* complex in the plasma membrane to activate PIP5K1*α*, which is required for PLC-*γ*1 activation leading to keratinocyte differentiation.

## 1. Introduction

The integral role of calcium in keratinocyte differentiation and in the regulation of epidermal functions is well established [1–8]. Disturbance of calcium homeostasis and aberrant keratinocyte differentiation and/or epidermal barrier dysregulation have been implicated in various skin pathologies [9–15], but the mechanism is not fully understood. We have previously proposed a possible signaling pathway that involves the extracellular calcium-induced recruitment of PI3K, PLC-*γ*1, and PIP5K1*α* leading to keratinocyte differentiation [16–19].

Epidermal keratinocytes have been shown to go through a terminal differentiation phase as they migrate from stratum basale to stratum corneum [20]. These in vitro differentiation processes can be reproduced by culturing keratinocytes in media with high calcium concentration, and these events closely resemble those of terminal differentiation of keratinocytes in vivo [1, 3, 5, 6]. In culture, keratinocytes morphologically resemble basal epidermal cells and fail to differentiate in calcium concentrations less than 0.03 mM. But at calcium concentrations exceeding 0.1 mM, keratinocytes morphologically resemble suprabasal epidermal cells and achieve the capability to differentiate [2]. Keratinocyte differentiation involves an intricate pathway involving cell-cell adhesion [3] and raised intracellular free calcium level [21] as a consequence of calcium release from internal stores and calcium influx through calcium channels in the plasma membrane [22], both of which are triggered by the increased extracellular calcium level. Extracellular calcium brings about the redistribution of cell adhesion molecules from the cytosol to the plasma membrane and the formation of intercellular contacts [3, 23]. The elevation of extracellular calcium results in an acute and sustained rise of intracellular calcium in the keratinocytes [24, 25] in the presence of PIP5K1*α* [19], both of which are prerequisites for calcium-induced keratinocyte differentiation [19, 26].

The role of phosphoprotein phosphatases and protein kinases in cellular functions and signal transduction pathways is well-known [27, 28]. Phosphoprotein phosphatase 1 (PP1) is a major serine/threonine phosphatase of the phosphoprotein phosphatase family [29]. Among the four major protein serine/threonine phosphatases, PP1 and PP2A are the two main ones that regulate diverse cellular events [30–32]. Other members of this superfamily include PP2B (calcineurin, PP3) [33]. PP1 is composed of the catalytic subunit and a wide variety of targeting/regulatory subunits [31, 32]. Four isoforms of the catalytic subunit of PP1, namely, *α*, *γ*1, *γ*2, and *δ*, have similar properties [33] and are expressed in mammalian tissues [34–38]. The intricate balance of phosphorylation by kinases and dephosphorylation by phosphatases is essential for maintaining signal transduction networks in cells [39]. PP1 is essential in many cellular events including calcium signaling [40] and has been regarded as a key player in the regulation of inositol-1,4,5-triphosphate receptor- (IP3R-) dependent calcium signaling [41]. IP3R acts as a calcium channel that releases calcium stored in the endoplasmic reticulum to the cytoplasm [42]. In one study, PP1 inhibition by okadaic acid reduced calcium levels [43]. Knockdown of PIP5K1*α* blocks calcium-induced PIP_2_ and IP_3_ production, intracellular calcium rise, and keratinocyte differentiation. PIP5K1*α* has been shown to be activated by dephosphorylation via protein kinase C (PKC) activated PP1. The possible role of PP1 in the stimulation of PIP5K1*α* synthesis by calcium to sustain IP_3_ production and keratinocyte differentiation cannot be overlooked. In light of this, we hypothesized that PP1 may play a role in the regulation of calcium release, thereby affecting the extracellular calcium-induced activation of PIP5K1*α* and having an impact on the calcium-induced PIP_2_ synthesis, PLC-*γ*1 activation, and IP_3_ production, eventually leading to keratinocyte differentiation. To test this hypothesis, we examined the effect of PP1 knockdown on keratinocyte differentiation induced by calcium.

## 2. Materials and Methods

### 2.1. Cell Culture

Human keratinocytes were isolated from neonatal human foreskins from the Department of Urology of The Second Xiangya Hospital of Central South University as done previously [44]. Primary cultures were established in serum-free medium (medium 154CF with human keratinocyte growth supplement, Cascade Biologics, Portland, OR) containing 0.07 mM calcium and second passage human keratinocytes were plated in serum-free medium containing 0.03 mM calcium and used in further experiments.

### 2.2. Small Interfering RNA Transfection

Keratinocytes with 10% confluence were transfected with small interfering RNA (siRNA) for E-cadherin, *β*-catenin, PIP5K*α*, or negative control (ON-TARGET plus*™* siRNA, Dharmacon, Chicago, IL) at a concentration of 100 nM using TransIT-siQUEST transfection reagent (Mirus, PanVera Corp., Madison, WI) at a dilution of 1 : 750 in accordance with the manufacturer's protocol.

### 2.3. Cell Lysate Preparation, Western Analysis, and Coimmunoprecipitation

Total cell lysates were isolated, for which PBS containing 2% SDS, complete protease inhibitors (Roche Applied Science), and 4-(2-aminoethyl) benzenesulfonyl fluoride (EMD Biosciences, CA) was used. Plasma membrane lysates were isolated using Mem-PER Eukaryotic Membrane Protein Extraction Reagent Kit (Pierce Biotechnology, Inc., Rockford, IL). The bicinchoninic acid (BCA) Protein Assay Kit (Pierce Biotechnology, Inc.) was used to measure the protein concentration of the lysate. Equal amounts of protein were electrophoresed, reducing SDS-PAGE, and electroblotted onto polyvinylidene fluoride (PVDF) membranes (Immobilon-P, 0.45 *μ*M, Millipore). After incubation in blocking buffer (100 mM Tris base, 150 mM NaCl, 5% nonfat milk, and 0.5% Tween 20), the blot was incubated overnight at 4°C with primary antibodies: polyclonal antibodies against human PIP5K1*α*, PLC-*γ*1, E-cadherin, p120-catenin, or transglutaminase 1 (Santa Cruz Biotechnology, Inc., CA) at a dilution of 1 : 200, monoclonal antibody against human *β*-catenin (Santa Cruz Biotechnology, Inc., CA) at a dilution of 1 : 200, antibody against PP1*α*, *β*, and *γ* (Santa Cruz Biotechnology, Inc., CA), polyclonal antibody against keratin 1 or keratin 5 (Covance Research Products, Inc., PA) at a dilution of 1 : 10000, monoclonal antibody against human involucrin (Sigma Aldrich Corporation, MO) at a dilution of 1 : 2000, polyclonal antibody against human p85 (Upstate Biotechnology, Inc., NY) which is the regulatory subunit of class Ia PI3K at a dilution of 1 : 1000, and monoclonal antibodies against human integrin 2 (plasma membrane marker), BIP (endoplasmic reticulum marker), or GM130 (cis-Golgi marker) at a dilution of 1 : 250 (BD Bioscience, CA). Then, the membranes were washed a few times and then incubated for 1 hour with anti-IgG secondary antibody conjugated to horseradish peroxidase (Amersham Biosciences Corp., NJ) in the blocking buffer. After another series of washes, bound antibody complexes were visualized using the Supersignal Ultra Chemiluminescent Kit (Pierce Biotechnology, Inc.) and exposed to X-ray film. To analyze protein complex formation at the plasma membrane by coimmunoprecipitation, equal amounts of plasma membrane protein (500 *μ*g) extracted with Mem-PER Eukaryotic Membrane Protein Extraction Reagent Kit were incubated with 2 *μ*g polyclonal antibody against E-cadherin for 1 hour at room temperature or overnight at 4°C and then with 20 *μ*L UltraLink Immobilized Protein A/G (Pierce Biotechnology, Inc.) for one hour at 4°C. After a series of washes, western analysis was done against PIP5K1*α*.

### 2.4. PIP5K1*α* Activity Assay

PIP5K1*α* activity was determined in accordance with the method as previously described by Chong et al. [45]. This method detects PIP_2_ formation from PI4P. The cells in 100 mm dishes were washed thrice with ice-cold PBS and extracted in Kinase Buffer (25 mM Tris/HCl, pH 7.4, 5 mM MgCl_2_, 1 mM EDTA, 0.1 mM EGTA, 1 mM dithiothreitol, 150 mM NaCl, 10% glycerol, 1% NP-40, AEBSF, Roche complete protease inhibitors, and Roche Phosphatase Inhibitor Cocktail Tablets). PIP5K1*α* was immune-precipitated from the lysate containing 500 *μ*g protein using 2 *μ*g polyclonal antibody against PIP5K1*α* for 1 hour at 4°C and then 20 *μ*L UltraLink Immobilized Protein A/G for 1 hour at 4°C. After a few washes, 10 *μ*L conjugated beads were mixed with 50 *μ*L Kinase Buffer containing sonicated phospholipids (70 *μ*M PI4P and 35 *μ*M phosphatidylserine, final concentrations) and preincubated for 15 min at 25°C. Reactions were started by the addition of 20 *μ*M [*γ*-^32^P] ATP (1 *μ*Ci/assay). After incubation for 5 min at 25°C, 0.3 mL of methanol, 1 N HCl (1 : 1, v/v) was added and extracted with 0.25 mL of chloroform. The organic layer was dried, resuspended in chloroform, and chromatographed on oxalate-pretreated Silica Gel 60 plates. Plates were developed in chloroform: methanol: 2.5 N ammonium hydroxide at a ratio of 9 : 7 : 2 by volume and then the [^32^P]-labeled products were observed by autoradiography.

### 2.5. Statistical Analysis

Data were analyzed by Student's paired *t*-test for data comparison of paired samples and analysis of variance with a post hoc test for more groups using SPSS 16.0. The data are presented as mean ± SD of three separate experiments. The results are expressed as percentages of the values in the control lane (presence of 0.03 mM Ca^2+^ and control siRNA). A probability (*p*) value of less than 0.05 was considered statistically significant (^*∗*^ *p* < 0.05).

## 3. Results

### 3.1. Calcium Induces PIP5K1*α* Dephosphorylation

**Figure 1:**
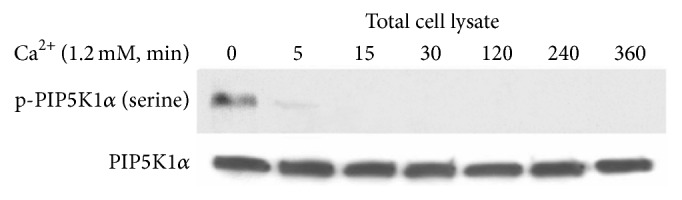
Calcium induces PIP5K1*α* dephosphorylation. Human keratinocytes cultured with 1.2 mM and harvested at time points indicated and analyzed by western analysis.

To determine the upstream regulatory mechanism by which calcium activates PIP5K1*α* and its role in keratinocyte differentiation, human keratinocytes were treated with 1.2 mM calcium and harvested at 0, 5, 15, 30, 120, 240, and 360 minutes. The plasma membrane and total cell lysates were isolated. The results show that calcium treatment caused increased serine dephosphorylation of PIP5K1*α* as early as 5 minutes (Figure 1).

### 3.2. Knockdown of Phosphoprotein Phosphatase 1 (PP1) Blocks Calcium-Induced PIP5K1*α* Dephosphorylation, Calcium-Induced PIP5K1*α* Activity, and Calcium-Induced Keratinocyte Differentiation

**Figure 2:**
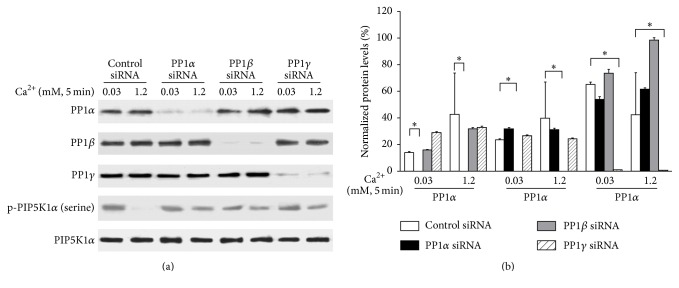
Knockdown of PP1 blocks calcium-induced PIP5K1*α* dephosphorylation. Cultured human keratinocytes were treated with PP1*α* siRNA, PP1*β* siRNA, and PP1*γ* siRNA for 72 hours and then with calcium for 5 min. (a) Cells were harvested and protein levels of PP1*α*, PP1*β*, and PP1*γ* were determined by western analysis. (b) Data expressed are mean ± SD of three separate experiments, ^*∗*^ *p* < 0.05.

**Figure 3:**
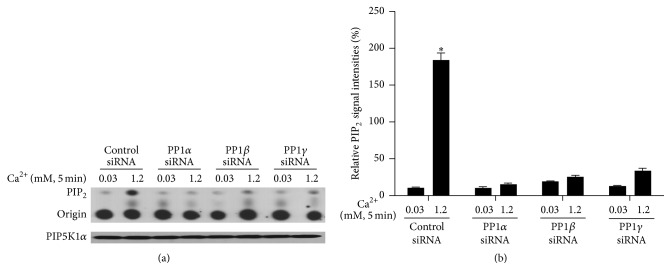
Knockdown of PP1 blocks calcium-induced PIP5K1*α* activity. Cultured human keratinocytes were treated with siRNA for PP1 for 72 hours and then with calcium for 5 min. Cells were harvested and total cell lysates were isolated for PIP5K1*α* activity assay. (a) The autoradiograph shown is from a representative experiment repeated thrice with three separate siRNA treatments. (b) The PIP_2_ signal intensities were quantitated by Image Pro Plus Software and normalized to band intensities of PIP5K1*α* in the corresponding western blot. Results are expressed as percentages of the values in the control lane (the presence of 0.03 mM calcium and control siRNA). Data expressed are mean ± SD of three separate experiments, ^*∗*^ *p* < 0.05 (significantly different from the control in the presence of 0.03 mM calcium and siRNA).

To determine whether PP1 plays a role in calcium-induced keratinocyte differentiation, normal human keratinocytes were pretreated with siRNA for PP1*α*, *β*, and *γ* for 3 days and then exposed to 1.2 mM calcium for 24 hours. Cells were harvested and total lysates were isolated. Protein levels of PP1*α*, *β*, and *γ* were determined to assess knockdown efficiency. The results show specific knockdown of the different PP1s by the specific mRNAs (Figure 2). No compensation by the other PP1 isoforms was seen when one was knocked down. To determine the role of PP1 knockdown in calcium-induced PIP5K1*α* activation, cultured normal human keratinocytes were treated with siRNA for PP1*α*, *β*, and *γ* for 3 days and then with calcium for 5 min. Cells were harvested and total cell lysates were isolated for PIP5K1*α* activity assay by measuring PIP_2_ formation. The results showed that knockdown of any of the three isoforms of PP1 blocked calcium-induced PIP5K1*α* activation (Figure 3). The finding that any one PP1 isoform has identical effects on inhibiting PIP5K1*α* might be because all three subunits of PP1 form a complex and blocking of any one would disrupt the complex.

**Figure 4:**
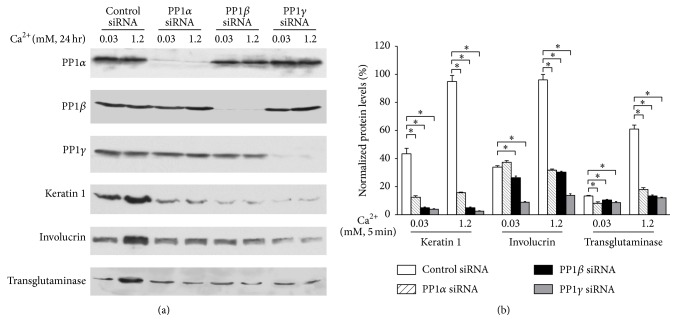
Knockdown of PP1 blocks calcium-induced keratinocyte differentiation. Cultured human keratinocytes were treated with PP1*α* siRNA, PP1*β* siRNA, and PP1*γ* siRNA for 72 hours and then in calcium for 5 min. Cells were harvested and total cell lysates were isolated. (a) The protein levels of PP1*α*, PP1*β*, PP1*γ*, keratin 1, involucrin, and transglutaminase were determined by western analysis. (b) Data expressed are mean ± SD of three separate experiments, ^*∗*^ *p* < 0.05.

Protein levels of differentiation markers, keratin 1 (early differentiation marker), involucrin, and transglutaminase (mid-differentiation markers), in normal human keratinocytes in which PP1 was reduced were evaluated (Figure 4). The results showed that calcium-induced expression of all of these markers was blocked. These data indicate that PP1 is required for calcium-induced keratinocyte differentiation.

### 3.3. Calcium Induces PP1 Recruitment to E-Cadherin in the Plasma Membrane of Human Keratinocytes

**Figure 5:**
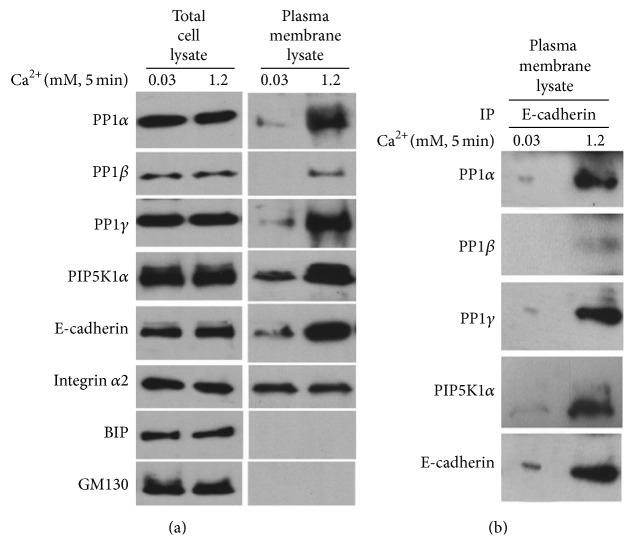
Calcium induces PP1 recruitment to E-cadherin in the plasma membrane. (a) Cultured human keratinocytes were treated with 1.2 mM calcium for 5 min. Cells were harvested and the protein levels of PP1*α*, PP1*β*, PP1*γ*, PIP5K1*α*, E-cadherin, integrin *α*2 (plasma membrane marker), BIP (endoplasmic reticulum marker), and GM130 (cis-Golgi marker) in total cell lysate and plasma membrane lysate were determined by western analysis. The results are from a representative experiment that was repeated three times. (b) The total cell lysates and the plasma cell lysates were isolated and analyzed by immunoprecipitation (IP) with antibody against E-cadherin followed by western analysis with antibodies against PP1*α*, PP1*β*, PP1*γ*, PIP5K1*α*, and E-cadherin.

In our previous studies [19], we showed that the role of PIP5K1*α* recruited by the E-cadherin-catenin complex to the plasma membrane in calcium-induced keratinocyte differentiation provides the substrate PIP_2_ for both PI3K and PLC-*γ*1. This pathway is critical for calcium-induced generation of the second messengers IP_3_ and intracellular calcium and keratinocyte differentiation. To determine whether high calcium induces recruitment of PP1 to the E-cadherin-*β*-catenin-PIP5K1*α* complex in the plasma membrane, human keratinocytes were treated with 1.2 mM calcium for 5 minutes, and total cell lysates and plasma membrane lysates were isolated. The protein levels of E-cadherin, PIP5K*α*, and PP1 were determined by western analysis. The results showed that calcium exposure for 5 minutes increased the expression of E-cadherin, PIP5K*α*, and PP1 in the plasma membrane lysate, but the expression levels of these proteins were not changed in the total cell lysate. Of the organellar markers tested, only the plasma membrane marker integrin *α*2 immunoreacted with the plasma membrane lysate (Figure 5(a)). This further documents the purity of the plasma membrane lysate preparation. To demonstrate an interaction of PP1 with E-cadherin in the plasma membrane, the plasma membrane lysates were immunoprecipitated with E-cadherin antibody. Western analysis of the immunoprecipitate with antibodies against PP1 or PIP5K*α* showed their association with E-cadherin. Taken together, our results indicate that PP1 is recruited to the calcium-dependent E-cadherin-catenin-PIP5K1*α* complex in the plasma membrane to activate PIP5K1*α*, which is required for PLC-*γ*1 activation for keratinocyte differentiation (Figure 5(b)).

### 3.4. Inhibition of PI3K, PLC, or PKC Activity Blocks Calcium-Induced PIP5K1*α* Dephosphorylation and Calcium-Induced PIP5K1*α* Activity

**Figure 6:**
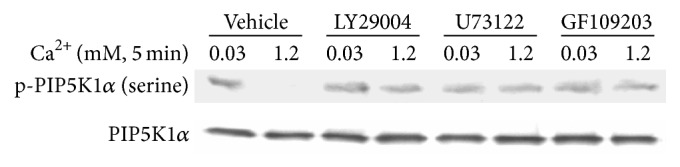
Inhibition of PI3K, PLC, or PKC activity blocks calcium-induced PIP5K1*α* dephosphorylation. Cultured human keratinocytes were treated with inhibitors for PI3K, PLC, and PKC for 72 hours and then with calcium for 5 min. Cells were harvested and then analyzed by western analysis.

**Figure 7:**
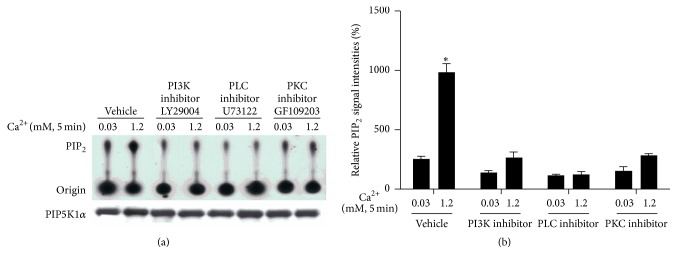
Inhibition of PI3K, PLC, or PKC activity blocks calcium-induced PIP5K1*α* activity. Cultured human keratinocytes were treated with inhibitors for PI3K, PLC, and PKC for 72 hours and then with calcium for 5 min. Cells were harvested and total cell lysates were isolated for PIP5K1*α* activity assay. (a) The autoradiograph shown is from a representative experiment repeated thrice with three separate siRNA treatments. (b) The PIP_2_ signal intensities were quantitated by Image Pro Plus Software and normalized to band intensities of PIP5K1*α* in the corresponding western blot. Results are expressed as percentages of the values in the control lane (the presence of 0.03 mM calcium and control siRNA). Data expressed as mean ± SD of three separate experiments, ^*∗*^ *p* < 0.05 (significantly different from the control in the presence of 0.03 mM calcium and siRNA).

Cultured human keratinocytes were treated with inhibitors for PI3K (LY29004, 10 *μ*M), PLC (U73122, 3 *μ*M), or PKC (GF109203, 2 *μ*M) for 72 hours and then with calcium for 5 min. Cells were harvested and total cell lysates were isolated for the PIP5K1*α* activity assay. The results showed that inhibition of PI3K, PLC, or PKC activity blocked calcium-induced PIP5K1*α* dephosphorylation and calcium-induced PIP5K1*α* activation (Figures 6 and 7).

### 3.5. Knockdown of E-Cadherin/Catenins or PLC-*γ*1 Blocks Calcium-Induced PIP5K1*α* Dephosphorylation and Calcium-Induced PIP5K1*α* Activation

**Figure 8:**
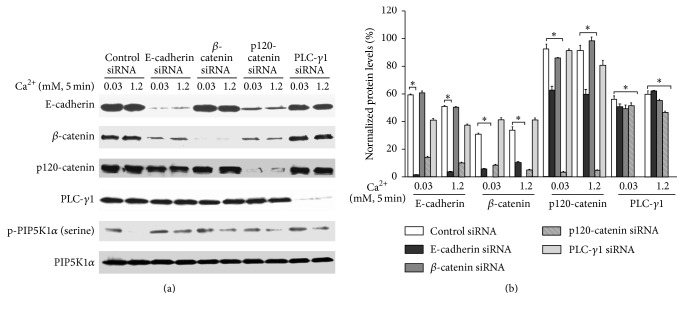
Knockdown of E-cadherin/catenins or PLC-*γ*1 blocks calcium-induced PIP5K1*α* dephosphorylation. Cultured human keratinocytes were treated with E-cadherin siRNA, *β*-catenin siRNA, p120 siRNA, and PLC-*γ*1 siRNA for 72 hours and then in calcium for 5 min. Cells were harvested and total cell lysates were isolated. The protein levels of E-cadherin, *β*-catenin, p120, PLC-*γ*1, p-PIP5KI*α* (serine), and PIP5KI*α* were analyzed by western analysis. Data expressed are mean ± SD of three separate experiments, ^*∗*^ *p* < 0.05.

**Figure 9:**
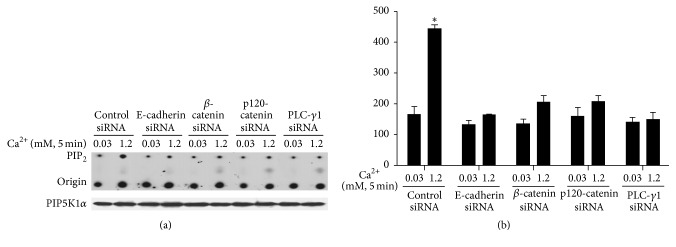
Knockdown of E-cadherin/catenins or PLC-*γ*1 blocks calcium-induced PIP5K1*α* activity. Cultured human keratinocytes were treated with siRNA for E-cadherin, *β*-catenin, p120, and PLC-*γ*1 for 72 hours and then with calcium for 5 min. Cells were harvested and total cell lysates were isolated for PIP5K1*α* activity assay. (a) The autoradiograph shown is from a representative experiment repeated thrice with three separate siRNA treatments. (b) The PIP_2_ signal intensities were quantitated by Image Pro Plus Software and normalized to band intensities of PIP5K1*α* in the corresponding western blot. Results are expressed as percentages of the values in the control lane (the presence of 0.03 mM calcium and control siRNA). Data are expressed as mean ± SD of three separate experiments, ^*∗*^ *p* < 0.05 (significantly different from the control in the presence of 0.03 mM calcium and siRNA).

Cultured human keratinocytes were treated with siRNA for E-cadherin, *β*-catenin, p120, or PLC-*γ*1 for 3 days and then exposed to calcium for 5 min. Cells were harvested and total cell lysates were isolated. The protein levels of E-cadherin, *β*-catenin, p120, PLC-*γ*1, p-PIP5KI*α* (serine), and PIP5KI*α* were analyzed by western analysis. The results showed that knockdown of E-cadherin/catenin or PLC-*γ*1 blocked calcium-induced PIP5K1*α* dephosphorylation (Figure 8). To determine whether high calcium induces PIP5K1*α* activity, cultured human keratinocytes were treated with 1.2 mM calcium for 5–360 min (Figure 9). Cells were harvested and total cell lysates were isolated for PIP5K1*α* activity assay. Consistent with an increase in PIP_2_ level, calcium induced PIP5K1*α* activity in a time-dependent manner. Then, we wanted to know whether E-cadherin, *β*-catenin, and PLC-*γ*1 were required for the activation of PIP5K1*α*. To address this issue, E-cadherin, *β*-catenin, or PLC-*γ*1 was knocked down by siRNA before calcium treatment. Cells were harvested, and total cell lysates were isolated for PIP5K1*α* activity assay. The results showed that E-cadherin or *β*-catenin knockdown blocked calcium-induced PIP5K1*α* activation. In addition, knockdown of PLC-*γ*1, the downstream effector of PIP5K1*α*, in these cells blocked upstream dephosphorylation and activation of PIP5K1*α* induced by calcium. These data indicate that E-cadherin, *β*-catenin, and PLC-*γ*1 are required for calcium-induced PIP5K1*α* activation.

## 4. Discussion

Protein phosphatase and kinase enzymes together with changes in intracellular calcium have been shown to possess significant roles in regulation of cell differentiation. We know that PIP5K1*α* activation is an important step in calcium-induced keratinocyte differentiation. In this study, we show that PP1 plays a role in calcium-induced keratinocyte differentiation, and it also mediates calcium-induced PIP5K1*α* activation and dephosphorylation. Under high calcium concentration, PP1 is recruited to the E-cadherin-*β*-catenin-p120 catenin complex. This study further shows that the signaling pathway involves the E-cadherin/*β*-catenin/p120 catenin complex, PKC, PLC-*γ*1, and PI3K, via PIP5K1*α* activation. It is possible, therefore, to speculate that high calcium induces formation of the E-cadherin-*β*-catenin-p120 catenin complex and subsequently recruits PIP5K1*α*, PI3K, and PP1.

**Figure 10:**
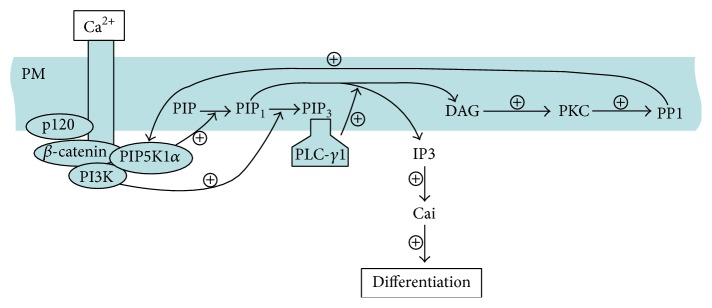
The proposed model. A proposed model for the signaling pathway of calcium-induced keratinocyte differentiation. In high calcium conditions, PP1 is recruited to the calcium-dependent E-cadherin-*β*-catenin-PIP5K1*α* complex in the plasma membrane to activate PIP5K1*α*, which increases the synthesis of the substrate PIP_2_ for both PLC-*γ*1 and PI3K. PLC-*γ*1 hydrolyzes PIP_2_ to IP_3_, which in turn increases calcium concentration leading to keratinocyte differentiation.

The model for this process is as follows. Calcium recruits the E-cadherin-*β*-catenin-p120-catenin (p120) complex to the plasma membrane (Figure 10). The complex then recruits and activates PI3K leading to PIP_3_ accumulation, which recruits and activates PLC-*γ*1. PLC-*γ*1 hydrolyzes PIP_2_ to IP_3_ to trigger keratinocyte differentiation by stimulating calcium release from the endoplasmic reticulum and Golgi thereby increasing the intracellular calcium level. PIP5K1*α* is also recruited to the E-cadherin-*β*-catenin complex where it is activated by PLC-*γ*1/PKC dependent PP1 via a feed-forward mechanism to continuously supply the substrate PIP_2_ for both PLC-*γ*1 and PI3K. In other words, PP1 is recruited to the calcium-dependent E-cadherin-*β*-catenin-PIP5K1*α* complex in the plasma membrane to activate PIP5K1*α*. This increases the synthesis of the substrate PIP_2_ for both PLC-*γ*1 and PI3K. PLC-*γ*1 hydrolyzes PIP_2_ to IP_3_. This increases calcium concentration, which leads to keratinocyte differentiation.

Previous studies have shown the roles of PIP5K1*α*, PLC-*γ*1, and PI3K in mediating calcium-induced differentiation [16–19]. PIP5K1*α* converts PIP to PIP_2_, and PI3K converts PIP_2_ to PIP_3_. This PIP_3_, in turn, activates PLC-*γ*1, which enhances the hydrolysis of PIP_2_ to IP_3_ and DAG. In the present study, we further explore the role of PP1 in calcium-induced differentiation via inducing activation and dephosphorylation of PIP5K1*α*.

There are two main ways by which calcium utilizes PP1 for its role in keratinocyte differentiation. The first one is as follows: calcium binds to E-cadherin, which recruits PP1 to the cell membrane. The second one could be the signaling pathway in which DAG activates PKC, which in turn activates PP1 that further brings about the dephosphorylation and activation of PIP5K1*α*. Our previous studies have shown that the activation of PIP5K1*α* plays an important role in differentiation of keratinocytes. The present study shows the role of PP1 in keratinocyte differentiation, also via PIP5K1*α* activation. This could be one novel addition to the mechanism by which calcium induces keratinocyte differentiation.

Intracellular calcium rise is necessary for keratinocyte differentiation [26]. The transient rise of intracellular calcium is not enough for keratinocyte differentiation, possibly due to the activation of PLC-*β* [39], whereas the sustained rise in intracellular calcium is required to induce differentiation possibly via activating PLC-*γ*1 [26]. Sustained increased levels of intracellular calcium can be achieved by the increase in the extracellular calcium level [24, 25] via the aforementioned pathway and store-operated calcium channels [46–48]. The roles of PIP_2_ and calcium in activating store-operated calcium entry have been put forward in a study in platelets [46] and that of PLC-*γ*1 has been put forward for the same in studies in liver cells [47] and keratinocytes [48]. PIP_2_ activates the downstream signaling leading to differentiation. High extracellular calcium concentration leads to PI3K recruitment to the E-cadherin-catenin complex [18, 49] and also the recruitment of PIP5K*α* [19] via *β*-catenin.

Various studies have shown conflicting results regarding the role of PP1 in tumor progression. A study by Kohno et al. shows the presence of PPP1R3 gene encoding the PP1 regulatory subunit in myriad human cancers, and there is a possibility of this gene being a tumor suppressor gene [50] because PP1*α* can dephosphorylate and activate the tumor suppressor gene, pRB [51]. In contrast, other studies depict PP1*α* as having an oncogenic effect, brought about by the dephosphorylation of breast and ovarian tumor suppressor protein BRCA1. Furthermore, the overexpression and/or increase in PP1 activity is associated with accelerated growth of malignant cells [52, 53]. Our present study shows that PP1 mediates calcium-induced differentiation in keratinocytes, suggesting the possible role of PP1 in tumor suppression. According to the results of our previous experiments, we found that the staining of the intracellular protein in keratinocytes is as sensitive as western blotting (Figure 4), so PIP5K1*α* staining on keratinocytes was not performed. In contrast to our findings, a study by Hsu et al. has shown that PP1 plays a role in tumorigenesis and progression of oral squamous carcinoma cells [54]. This study also shows that different oral squamous carcinoma cell lines have various levels of PP1*α*, which may define the rate of proliferation. The higher the PP1*α* level, the faster the proliferation. Knockdown of PP1*α* inhibits proliferation [54]. We believe that the differences between our results and the study by Hsu et al. arise, because of the different localization of PP1 distribution and the influence of various factors including calcium in the regulation of PP1 distribution [55, 56]. PP1 is localized in the plasma membrane, nucleus, and cytoplasm and has distinct site-specific roles [55, 57], but further localization studies should be done to explain the differences in the regulation and function of PP1.

In the present study, calcium recruits PP1 to the plasma membrane and aids in cell differentiation. Our previous study had shown that calcium has a protective role against oral squamous cell carcinoma [58]. Further research has to be done on the possible impact of the localization of PP1 on malignancy. But, only a very small fraction of PP1 would likely be recruited to the plasma membrane. PP1 is in over a hundred different complexes throughout the cell and any particular pathway will only involve a particular complex, which represents only a small pool of total cellular PP1.

Few studies have studied the effects of the knockdown of the components of the E-cadherin complex, to effects on keratinocyte marker expression [59, 60].

## 5. Conclusion

Phosphoprotein phosphatase 1 is required for calcium-induced PIP5K1*α* activation and keratinocyte differentiation.

## Abbreviations

DAG: Diacylglycerol
IP_3_: Inositol-1,4,5-trisphosphate
IP_3_R: Inositol-1,4,5-trisphosphate receptor
PI3K: Phosphatidylinositol-3-kinase
PIP_2_: Phosphatidylinositol-4,5-bisphosphate
PIP_3_: Phosphatidylinositol-3,4,5-triphosphate
PIP5K1*α*: Phosphatidylinositol-4-phosphate-5-kinase type 1*α*
PKC-*α*: Protein kinase C-*α*
PLC-*γ*1: Phospholipase C-*γ*1
PP1: Phosphoprotein phosphatase type 1
PP2A: Phosphoprotein phosphatase type 2A
PP2B: Phosphoprotein phosphatase type 2B.
